# Growth hormone combined with estrogen improves intrauterine adhesion fibrosis by downregulating endometrial microbial citraconic acid to target β-catenin protein

**DOI:** 10.1128/msystems.01692-24

**Published:** 2025-06-05

**Authors:** Yuhua Zeng, Yanfei Yang, Fei Zeng, Qing Feng

**Affiliations:** 1 Department of Health Management, The Third Xiangya Hospital, Central South University 12570, Changsha, Hunan, China; 2 Department of Obstetrics and Gynecology, Third Xiangya Hospital of Central South University 725459, Changsha, Hunan, China; Pacific Northwest National Laboratory, Richland, Washington, USA

**Keywords:** intrauterine adhesions, growth hormone, estrogen, endometrial microbiota, citraconic acid, β-catenin pathway

## Abstract

Intrauterine adhesions (IUAs) are a major cause of secondary infertility. This study aimed to investigate the therapeutic effects and mechanisms of combined recombinant rat growth hormone (rrGH) and estrogen therapy on endometrial fibrosis in IUA rats. Our findings revealed that IUA rats exhibited severe endometrial damage, heightened inflammatory responses, significant collagen deposition, and imbalances in various inflammatory and growth factors. However, these pathological changes were markedly improved following combined rrGH and estrogen treatment. Additionally, the endometrial microbial diversity in IUA rats was significantly reduced, and the combined therapy effectively promoted its restoration. Biochemical serum analysis showed that the combined therapy upregulated key reproductive hormone levels. Notably, the combined application of rrGH and estrogen partially restored GH receptor levels. TGF-β1, MMP9, and β-catenin were upregulated in the endometria of IUA rats, while the p-smad3/smad3 ratio was downregulated, and these key indicators were reversed after combined therapy. Furthermore, antibiotic treatment weakened the effects of combined therapy, indicating the role of endometrial microbiota in IUA. Molecular docking results revealed a high affinity between β-catenin and differential metabolites such as citraconic acid, suggesting their potential importance in regulating the β-catenin signaling pathway. In a TGF-β1-induced IUA cell model, we found that TGF-β1 treatment upregulated fibrosis-related protein expression but decreased β-catenin protein levels and stability. Citraconic acid intervention enhanced the effects of TGF-β1, while β-catenin overexpression inhibited these changes. In summary, the combined therapy targeting the β-catenin pathway through citraconic acid regulation alleviated endometrial fibrosis, offering a new approach to treating IUA.

**IMPORTANCE:**

Intrauterine adhesions (IUAs) are an important endometrial disease. Our study highlights the importance of the combination of recombinant rat growth hormone (rrGH) and estrogen in ameliorating endometrial damage and fibrosis, as well as promoting endometrial regeneration in IUA rats. In addition, our study emphasizes their important role in ameliorating microecological disturbances in the intrauterine environment and regulating serum metabolism. Our experiments also revealed for the first time that the combination of rrGH and estrogen may modulate endometrial microbes or influence the progression of IUA by promoting β-catenin expression, which is important for understanding the treatment of IUA disease. Our study provides new and important insights into the understanding and treatment of IUA disease.

## INTRODUCTION

Intrauterine adhesions (IUA) are a fibrotic disease caused by an injury in the basal layer of endometrium, which is the main cause of secondary infertility in pregnant women (1). Currently, the primary therapy for IUA treatment is hysteroscopic adhesiolysis to restore uterine shape, but various complementary treatments are also necessary to promote endometrial regeneration (2, 3). Estrogen was a commonly used hormone in the adjuvant therapy of IUA, but its efficacy was limited in severe cases (4). Moreover, the recent study suggested that postoperative estrogen therapy neither attenuated the incidence or severity of re-adhesions nor alleviated menstrual patterns (5). Growth hormone (GH) is an anterior pituitary hormone that stimulates the expression of insulin-like growth factor-I through the GH receptor (GHR)-JAK-STAT signal pathway, thus regulating cell proliferation and tissue growth (6, 7). Studies have shown that GH improves the pregnancy rate of thin endometrium patients receiving frozen embryo transfer by promoting endometrial cell proliferation and vascularization (8). In addition, GH also promotes the proliferation and movement of human endometrial glandular cells through the GHR-STAT3/5 pathway (9). GH might be a potential drug for the treatment of IUA. Therefore, whether the combination of GH and estrogen improves IUA more effectively deserves further exploration.

The pathogenesis of intrauterine adhesions exhibits a certain degree of correlation with the reproductive tract microbiome, which participates in the occurrence and progression of IUA by influencing immune functions and metabolism (10). It is reported that there are differences in endometrial microbiome between IUA patients and infertile patients without intrauterine lesions, and the potential variation of endometrial microbiome might lead to IUA (11). Clinical research implicated that IUA significantly reduces the percentage of *Lactobacillus* in 50% of affected patients, while significantly increasing the abundance of *Gardnerella* and *Prevotella*, which may exacerbate the severity of IUA and elevate the risk of recurrence (12). Notably, *Lactobacillus crispatus* promotes early postoperative recovery in patients with IUA by restoring vaginal microbial balance, inhibiting uterine inflammation and fibrosis (13). In addition, the microbiome was tightly associated with regulating systemic metabolism (14). Metabolomics studies indicated that the metabolism of amino acids and their derivatives and energy metabolism were significantly abnormal in the serum of IUA rats (15). Recent research has indicated that alterations in the intrauterine microbiome, specific uterine metabolites, and endometrial transcriptional characteristics are associated with IUA, and the formation of IUA can potentially be prevented through interventions targeting the underlying causes, microbial infections, and tenascin-like proteins (16). Hence, it is of great significance to confirm whether the combination of GH and estrogen improves the microbial structure of endometrium and serum metabolism.

Wnt signaling pathways can be divided into two types: non-canonical and canonical, with the canonical Wnt/β-catenin signaling believed to be related to various diseases, including cardiovascular disease, lung disease, liver disease, neurodegenerative disease, and tumors, among others (17). Notably, the unregulated Wnt/β-catenin pathway may contribute to the occurrence of fibrosis (18, 19). Studies by Yuan et al*.* (20) have indicated that bone marrow mesenchymal stem cells combined with estrogen inhibit fibrosis and promote endometrial regeneration through the Wnt/β-catenin pathway. In addition, Liu et al*.* (21) also reported that si-SNHG5-FOXF2 inhibits TGF-β1-induced fibrosis in endometrial stromal cells (ESCs) through the Wnt/β-catenin signaling pathway. As a core component of the Wnt signaling pathway, the β-catenin protein plays a crucial role in cell homeostasis, embryonic development, organogenesis, stem cell maintenance, cell proliferation, migration, differentiation, cell death, and the pathogenesis mechanisms of various diseases, including cancer (22). Its level is finely regulated by the β-catenin degradation complex consisting of proteins such as axin, adenomatous polyposis coli gene (APC), glycogen synthase kinase 3β (GSK-3β), and casein kinase 1α (CK1α), among others (23). Currently, it is still unclear whether GH and estrogen can treat IUA by targeting the β-catenin pathway. Further research is required for elucidation.

In this study, the IUA rat model was established to calculate the effect of GH combined with estrogen on endometrial repair by detecting endometrial pathological changes, inflammation, and fibrosis. Then, the effects of GH combined with estrogen on endometrial microbial structure and serum metabolism were described by 16S rRNA sequencing and non-targeted metabolomics. Finally, we evaluated the specific potential mechanisms of GH combined with estrogen in treating IUA in both the IUA rat model and TGF-β1-induced IUA cell model.

## MATERIALS AND METHODS

### IUA model construction

Female Sprague Dawley (SD) rats aged 10 weeks, weighing 200–250 g, were purchased from Hunan SJA Laboratory Animal Co., Ltd. Before the experiment, the rats underwent 1 week of adaptive feeding and were housed in a specific pathogen-free environment maintained at 22°C ± 1°C with a relative humidity of 50% ± 1%, under a 12 h light/dark cycle. The IUA model was established following previously described methods (24). Specifically, the estrous cycle was determined by vaginal smears, and the rats were anesthetized with 3% pentobarbital via intraperitoneal injection during the interphase. Under sterile conditions, a vertical incision (20 mm) was made longitudinally, and a 16-gauge needle was inserted and rotated to scratch the entire endometrial inner surface of both lateral walls until the uterine wall became rough and pale, while ensuring the uterine serosa remained intact. The uterus was washed with saline, and the uterine and abdominal incisions were sutured in layers.

### Experimental grouping and treatment

The rats were randomly divided into seven groups (*n* = 6): Control, IUA, Estrogen, recombinant rat growth hormone (rrGH), Estrogen + rrGH, Antibiotics, and Estrogen + rrGH + antibiotics groups. There was no intervention performed in the Control group. After two estrous cycles (about 9 days), the IUA rats in the Estrogen group were given 0.2 mg estrogen/kg of body weight by intragastric administration (25). In the rrGH group, IUA rats were subcutaneously injected with 0.15 U/100 g recombinant rat growth hormone (26). In the Estrogen + rrGH group, IUA rats were given both 0.2 mg estrogen/kg of body weight and 0.15 U/100 g of rrGH. In the Antibiotics group, IUA rats were given 1.14 mg of metronidazole and 0.29 mg of doxycycline hydrochloride tablets. In the Estrogen + rrGH + antibiotics group, IUA rats were given 0.2 mg estrogen/kg of body weight, 0.15 U/100 g of rrGH, 1.14 mg of metronidazole, and 0.29 mg of doxycycline hydrochloride tablets. The rats in the Control and IUA groups were treated with the same amount of normal saline. Metronidazole and doxycycline hydrochloride tablets were given orally twice daily, with doses converted based on the body surface area ratio between humans and rats (27). Estrogen and rrGH were injected subcutaneously every 2 days. The drug intervention lasted for two consecutive weeks. Then, the rats were sacrificed, and the endometrium and peripheral blood were collected for subsequent detection.

### Cell isolation and treatment

The endometrial tissue of SD rats was collected under aseptic conditions and washed with PBS three to four times. Then, the tissue was cut into pieces and digested with DMEM/F12 containing 0.1% collagenase IV at 37°C for 60 min. Subsequently, the cell suspension was filtered with a 40–100 µm filter to remove the incompletely digested tissue. Finally, the cell precipitates were collected by centrifuging at 1,000 rpm for 5 min and resuspended with DMEM/F12 containing 10% FBS. The obtained ESCs were identified by immunofluorescence (IF) and divided into six distinct groups: Control, IUA, oe-NC, oe-NC + citraconic acid, oe-β-catenin, and oe-β-catenin + citraconic acid groups. Cells in the control group were maintained under standard culture conditions without any treatment. In the IUA group, cells were exposed to 10 ng/mL of TGF-β1 for 72 h to mimic the IUA condition (28). In the oe-NC group, cells from the IUA group were transfected with an overexpression negative control (oe-NC) vector. In the oe-NC + citraconic acid group, cells from the IUA group were transfected with oe-NC and additionally treated with 10 mM citraconic acid. In the oe-β-catenin group, cells from the IUA group were transfected with an overexpression vector for β-catenin (oe-β-catenin). In the oe-β-catenin + citraconic acid group, cells from the IUA group were transfected with oe-β-catenin and further treated with 10 mM citraconic acid.

### Cell transfection

For gene silencing, cells were transfected with oe-β-catenin (HonorGene) using Lipofectamine 2000 (11668019, Invitrogen), following the manufacturer’s instructions. Oe-NC were used as controls. The normal culture medium was changed for continued culture after 6 h of transfection, and relevant detection was carried out 48 h later.

### Cell Counting Kit-8

After treatment for 24 h, the drug-containing medium was removed, and the ESCs were collected. A volume of 100 µL of medium containing 10 µL Cell Counting Kit-8 (CCK-8) (NU679, DoJindo, Japan) was added to each well and incubated at 37°C and 5% CO_2_ for 4 h. Then, the optical density (OD) at 450 nm was analyzed by a microplate reader (MB-530, HEALES, China).

### Enzyme-linked immunosorbent assay

A variety of enzyme-linked immunosorbent assay (ELISA) kits were utilized, strictly adhering to the standard operating procedures provided by the manufacturers, to quantify key biomarkers in rat endometrial tissues or peripheral blood. The biomarkers were detected using the following ELISA kits and their respective catalog numbers: TNF-α (CSB-E11987r), IL-4 (CSB-E04635r), IL-6 (CSB-E04640r), IL-10 (CSB-E04595r), GH receptor (GHR; LS-F50103), follicle-stimulating hormone (FSH; CSB-E06869r), luteinizing hormone (LH; CSB-E12654r), estradiol (E2; CSB-E05110r), progesterone (P; CSB-E07282r), testosterone (Tl CSB-E05100r), prolactin (PRLl CSB-E06881r). All ELISA kits, except for the GHR kit sourced from LSBio, USA, were sourced from Cusabio, China.

### Quantitative real-time PCR

Total RNA was extracted from endometrial tissue by the TRIzol method. The cDNA was obtained by mRNA Reverse Transcription Kit (CW2569, CWBIO, China) using RNA samples. UltraSYBR Mixture (CW2601) was applied to analyze the relative expression of the genes on the QuantStudio1 Real-Time PCR System (ABI, USA). The relative gene level was calculated by the 2^−ΔΔCt^ method with β-actin as the reference gene. The sequence of primers is shown in Table 1.

**TABLE 1:** Primer sequences

Gene	Sequence	Length (bp)
TNF-α	F 5′-CCCCTCTATTTATAATTGCACCT-3	167
	R 5-CTGGTAGTTTAGCTCCGTTT-3′	
IL-6	F 5′-TCACTATGAGGTCTACTCGG-3′	141
	R 5′-CATATTGCCAGTTCTTCGTA-3′	
IL-4	F 5′-GTACCGGGAACGGTATCCAC-3′	247
	R 5′-CTCAGTTCACCGAGAACCCC-3′	
IL-10	F 5′-AATAAGCTCCAAGACAAAGGT-3′	79
	R 5′-TCACGTAGGCTTCTATGCAG-3′	
Insulin-like growth factor-1	F 5′-GACCCGGGACGTACCAAAAT-3′	162
	R 5′-GAACTGAAGAGCGTCCACCA-3′	
Stromal cell-derived factor-1	F 5′-CCATGTCGCCAGAGCCAAC-3′	117
	R 5′-AATTTCGGGTCAATGCACACT-3′	
Thrombospondin-1	F 5′-GACAATTGCCCCTACAACCAC-3′	149
	R 5′-GTGTCCCTCTGATCCACGTT-3′	
β-Actin	F 5′-ACATCCGTAAAGACCTCTATGCC-3′	223
	R 5′-TACTCCTGCTTGCTGATCCAC-3′	

### Western blot

Total protein was extracted from endometrial tissues and ESCs using RIPA lysis buffer (AWB0136, Abiowell, China) and quantified by the BCA kit. The protein was separated on a 10% SDS-PAGE gel and then transferred to a PVDF membrane (Invitrogen, USA). The membrane was incubated with primary antibodies against smad3, p-smad3, MMP-9, β-catenin, GSK-3β, cyclin D1, AXIN, APC, CK1, β-TrCP, and β-actin overnight at 4°C. Then, the membrane was incubated with the secondary HRP goat anti-rabbit IgG antibody or HRP goat anti-mouse IgG antibody at room temperature for 2 h. ECL chromogenic solution (Chemiscope6100, Clinx, China) was used for development, and the gray value of protein bands was evaluated by Bio-Rad Quantity One version 4.62. The antibodies are shown in Table 2.

**TABLE 2:** Antibody information

Antibody type	Indicator	Cat. number	Species	Dilution ratio	Manufacturer, country
Primary antibodies	smad3	66516-1-Ig	Mouse	1:2,000	Proteintech, USA
	p-smad3	ab52903	Rabbit	1:2,000	Abcam, UK
	MMP-9	ab76003	Rabbit	1:5,000	Abcam, UK
	β-Catenin	51067-2-AP	Rabbit	1:10,000	Proteintech, USA
	GSK-3β	22104-1-AP	Rabbit	1:3,000	Proteintech, USA
	Cyclin D1	66009-1-Ig	Mouse	1:40,000	Proteintech, USA
	AXIN	16541-1-AP	Rabbit	1:1,000	Proteintech, USA
	APC	ab40778	Rabbit	1:5000	Abcam, UK
	CK1	16848-1-AP	Rabbit	1:1,000	Proteintech, USA
	β-TrCP	28393-1-AP	Rabbit	1:5,000	Proteintech, USA
	β-Actin	66009-1-Ig	Rabbit	1:5,000	Proteintech, USA
Secondary antibodies	HRP goat anti-mouse IgG	SA00001-1	Mouse	1:5,000	Proteintech, USA
	HRP goat anti-rabbit IgG	SA00001-2	Rabbit	1:6,000	Proteintech, USA

### Hematoxylin-eosin staining

Rat endometrial tissues were fixed with 4% paraformaldehyde, embedded in paraffin, and sectioned. The sections were deparaffinized by xylene and ethanol (100%–75%) and then immersed in distilled water for 5 min. Sections were stained sequentially with hematoxylin and eosin, rinsed in distilled water, and then dehydrated with gradient alcohol (95%–100%). Sections were removed and placed in xylene, then sealed with neutral gum and examined microscopically for endometrial damage.

### Masson’s trichrome staining

Rat endometrial tissues were fixed with 4% paraformaldehyde, embedded in paraffin, and sectioned. The sections were then deparaffinized by xylene and ethanol (100%–75%) and then immersed in distilled water for 5 min. An appropriate amount of nuclear staining solution was added dropwise to the sections, and the staining solution was rinsed off after 10 s. The sections were soaked in distilled water and PBS sequentially. They were then stained with an appropriate amount of plasma staining solution for 5 min, rinsed, and then stained with color separation solution for about 30 s. The sections were then washed in distilled water and PBS for 5 min. Then, the sections were stained with a staining solution for 1 min and rinsed with anhydrous ethanol. After the sections were dried, they were made transparent with xylene. The sections were sealed, and finally, the fibrosis of rat endometrial tissue was observed by microscope (BA410E, Motic, China).

### Immunohistochemistry

The sections were treated with 3% H_2_O_2_ for 10 min at room temperature to inactivate endogenous enzymes. After dilution of the antibodies using TE buffer pH (9.0), sections were incubated with primary antibodies against collagen I (1:50; 14695-1-AP, PTG), CK-18 (1:50; 10830-1-AP, PTG), vimentin (1:50; 10366-1-AP, PTG), and TGF-β1 (1:50; 21898-1-AP, PTG) at 4°C overnight, followed by incubation with anti-rabbit IgG antibody-HRP polymer (7074P2, CST, USA) at 37°C for 30 min. Finally, the sections were treated with diaminobenzidine (50 µL, ZLI-9017, ZSGBbio, China) for 1–5 min. Images were collected using a microscope.

### Immunofluorescence

Slides were prepared and fixed with 4% paraformaldehyde. After permeating with 0.3% Triton at 37°C for 30 min, the slides were blocked with 5% BSA at 37°C. Subsequently, the slides were incubated with primary antibodies against α-SMA (ab124964, Abcam, UK), collagen I (14695-1-AP, PTG, USA), CK-19 (10712-1-AP, PTG, USA), and vimentin (10366-1-AP, PTG, USA) at 4°C overnight, followed by incubation with CoraLite488-conjugated Affinipure Goat Anti-Rabbit IgG (SA00013-2, Proteintech, USA) at 37°C for 90 min. DAPI was applied to stain the nucleus. The images were obtained by a microscope.

### 16S rRNA sequencing

Genomic DNA was extracted from rat uterine lavage fluid using the HiPure bacterial DNA kit (D3146-02, Magen, China). The V3/V4 region of 16S rRNA was amplified using PrimeSTAR Max DNA Polymerase (R045Q, Takara, Japan) and 341F/805R primers (341F, 5′-CCTACGGGNGGCWGCAG-3′; 805R, 5′-GACTACHVGGGTATCTAATCC-3′). The 16S amplifiers of RNA were then sequenced using the high-throughput Illumina NovaSeq PE250 platform to obtain the original data. The raw data were quality assessed with Qiime 2 (Qiime2-2020.2), trained to obtain operational taxonomic units (OTUs) regarding the V3-V4 region of the genomic Silva138 99% clustered sequences. Species annotation of the OTUs was carried out by 16S sequencing and database comparisons, and analyses such as Alpha Diversity were performed by examining the distributions of species information and the abundance of the species. The box plots (R phyloseq package) and petal plots (Jenn: http://www.bioinformatics.com.cn/static/others/jvenn) (29) were plotted, respectively. The differences in the phylum levels and genera of microorganisms in the uterine lavage fluid were analyzed by LefSe (https://github.com/SegataLab/lefse). The relative abundance histogram of species and the heat map of genus abundance were drawn by R software (R phyloseq package).

### Untargeted metabolomics

The peripheral blood serum of rats was collected for metabonomic analysis. Every 100 µL sample was mixed with 300 µL cold acetonitrile and vortexed for 30 s before analysis. The mixture was deproteinized by centrifugation at 4°C (21,130 × *g*, 30 min), and 1 µL supernatant was injected into UPLC. LC-MS/MS analysis was carried out using MS (QExactivePlus, ThermoScientific), UHPLC system (1290, Agilent Technologies), and UPLC BEH Amide column (1.7 µm, 2.1 × 100 mm, Waters) combined with TripleTOF 6600 (Q-TOF, AB Sciex) and QTOF 6550. The mobile phase consists of 25 mM NH_4_OAc and 25 mM NH_4_OH (pH 9.75) (A) and acetonitrile (B) in water. The original data were collected and then analyzed by the MetaboAnalyst 5.0 platform (https://www.metaboanalyst.ca/).

### Co-immunoprecipitation

Cell proteins were extracted using RIPA lysis buffer. The cell lysates were then incubated overnight at 4°C with either β-catenin or rabbit IgG (B900610, Proteintech, USA) antibodies. To capture the immune complexes, 10 µL of protein A/G agarose beads was added to the sample, which was subsequently incubated at 4°C for 2 h. The agarose beads carrying the immune complexes were then washed three times with lysis buffer. After centrifugation at 3,000 rpm for 3 min, the residual agarose beads were heated with the sample buffer solution for 5 min. The resulting mixture was centrifuged again to collect the liquid for further analysis using western blot (WB) analysis.

### Molecular docking

The 2D structures of the metabolites, namely citraconic acid, itaconate, fumaric acid monomethyl ester, 2-methylmaleate, mesaconic acid, and 5-oxo-2-tetrahydrofuran carboxylic acid, were retrieved from PubChem and converted into 3D structures using Corina. Subsequently, the crystal structure of β-catenin was downloaded from the PDB database (https://www.rcsb.org). PyMOL was employed to remove water molecules, original ligands, and other non-protein entities from the crystal structure. The prepared metabolites and β-catenin underwent preprocessing with AutoDock Tools. This step encompassed hydrogenation to mimic the molecular state under physiological conditions, charge calculation to maintain charge balance during docking, and conversion of molecular structures to the PDBQT format required by AutoDock Vina. Within AutoDock Vina, docking parameters were configured. Docking calculations were performed separately for each metabolite, ensuring the generation of at least nine conformations per docking process. The conformation with the lowest binding energy was selected as the optimal docking conformation, representing the most stable binding mode between the metabolite and β-catenin. Finally, the optimal docking conformation was visually analyzed using PyMOL.

### High-performance liquid chromatography-tandem mass spectrometry

The sample analysis was performed using a Shimadzu Nexera UHPLC LC-30A system coupled with an AB SCIEX Triple TOF 5600+ system equipped with an ESI source. A sample volume of 3 µL was injected. The target analytes were separated using a T3 column (2.1 × 100 mm, 1.7 µm, Waters). The column temperature was maintained at 40°C, and the mobile phases consisted of 0.1% formic acid aqueous solution (phase A) and acetonitrile (phase B). The gradient elution started with an isocratic flow of 1% B (and 99% A) for 1.5 min, followed by a linear increase of B to 99% within 13 min and a 3.5 min hold period. The phase composition was restored to 1% B within 0.1 min and held for 5 min for column equilibration. The flow rate was set at 0.3 mL/min. The mass spectrometer operated in positive and negative ion information-dependent acquisition (IDA) modes. The instrument parameters were as follows: the source temperature was set at 550°C, gas 1 and gas 2 were maintained at 55 psi, the curtain gas was set at 35 psi, and the ion spray voltage was set at 5.5 kV in positive mode and −4.5 kV in negative mode. The full scan accumulation time was set at 150 ms, and each IDA experiment had an accumulation time of 45 ms. The mass range was set from *m*/*z* 60 to *m*/*z* 1,250, and the collision energy was set at 30 or −30 eV. Compounds with peak intensities greater than 100 cps were selected for further analysis. Data collection was performed using Analyst TF 1.6 software (SCIEX), and analysis was conducted using AB SCIEX OS software and MetaboAnalyst 5.0 for metabolomics deconvolution and pathway analysis.

### Statistical analysis

GraphPad Prism 9.0 was employed to analyze the data. All the experiments were repeated three times, and the data are shown as mean ± standard deviation. Student’s *t*-test and one-way analysis of variance were conducted to assess the statistical differences between two or more groups. Pearson correlation analysis was performed. *P* < 0.05 was considered as statistically significant.

## RESULTS

### rrGH combined with estrogen improves endometrial injury and fibrosis in IUA rats

**Fig 1:**
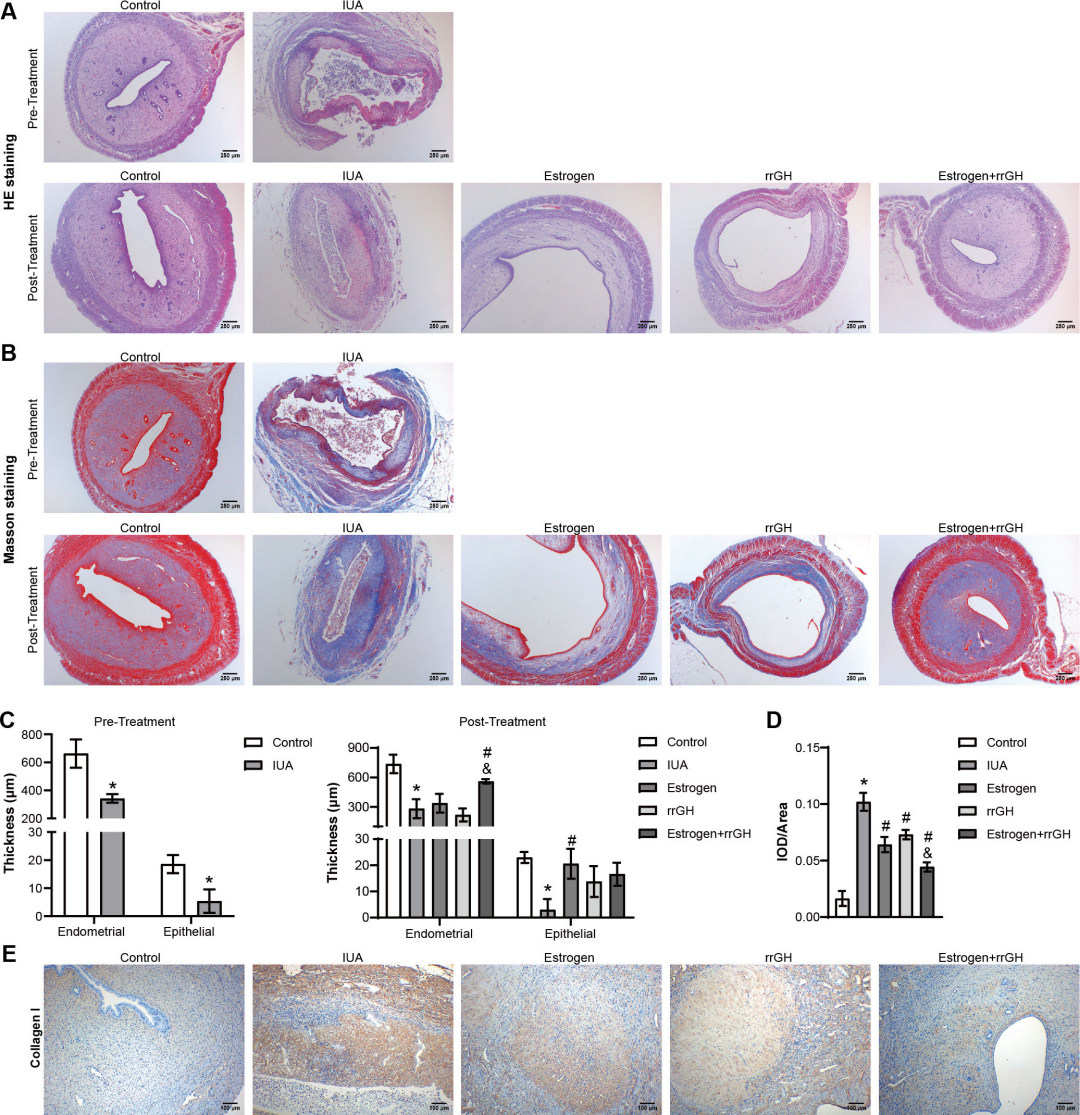
rrGH combined with estrogen improves endometrial injury and fibrosis in IUA rats. (**A and B**) HE and Masson staining of the uterus before (above) and after (below) treatment (scale bar: 250 µm). (**C**) The thickness of the endometrium and epithelium before (left) or after (right) treatment. (**D and E**) Immunohistochemistry staining analysis of collagen I expression in the endometrial tissue of IUA rats (scale bar: 100 µm). *P < 0.05 vs Control group, ^#^*P* < 0.05 vs IUA group, and ^&^*P* < 0.05 vs Estrogen group; *n* = 3.

First, an IUA rat model was established to investigate the effects of combined rrGH with estrogen on endometrial injury and fibrosis induced by IUA. Compared with the Control group, HE and Masson staining showed a decrease in endometrial glands, thinning of the endometrial and epithelial layers, and a significant increase in inflammation and collagen deposition, suggesting successful induction of endometrial injury and fibrosis in rats. These were significantly improved in the rrGH group, the Estrogen group, and the Estrogen + rrGH group after 2 weeks of intervention compared to the IUA group. Importantly, rats in the Estrogen + rrGH group showed increased endometrial glands, thickening of the endometrium and epithelial layers, and a significant reduction in inflammation and collagen deposition compared to the Estrogen group, with the Estrogen + rrGH group showing a more significant therapeutic effect (Fig. 1A through C). Further results showed that collagen I was upregulated in the IUA group compared to the Control group rats. In contrast, the rrGH group, the Estrogen group, and the Estrogen + rrGH group all showed a marked reduction in collagen I expression compared to the IUA group. Notably, the trend of upregulation of collagen I was more significant in the Estrogen + rrGH group compared to the Estrogen group (Fig. 1D and E). The above results suggest that the combined use of rrGH and estrogen significantly improves endometrial injury and fibrosis in IUA rats.

### rrGH combined with estrogen inhibits endometrial inflammation and promotes endometrial regeneration in IUA rats

**Fig 2:**
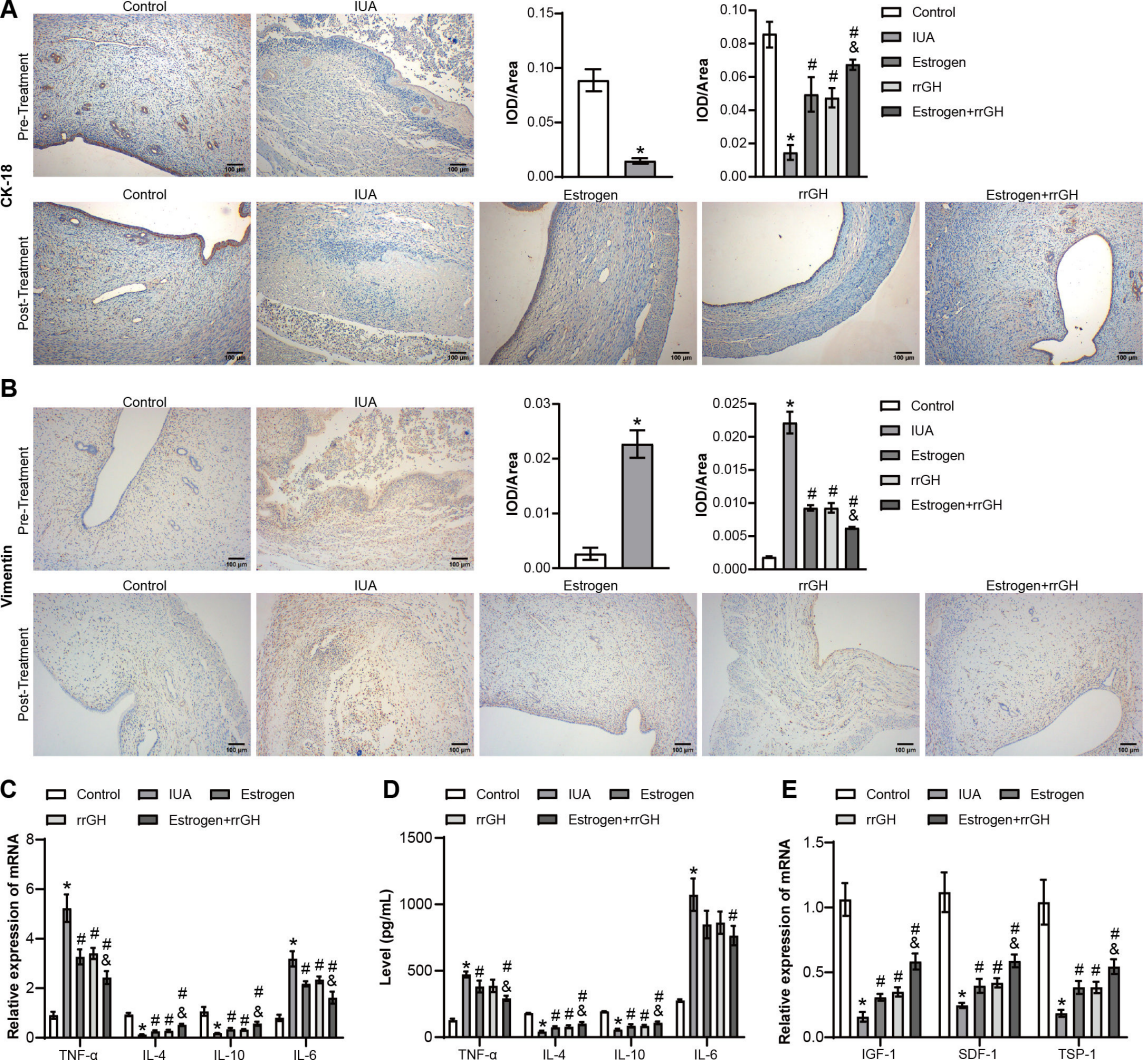
rrGH combined with estrogen inhibits endometrial inflammation and promotes endometrial regeneration in IUA rats. (**A and B**) IHC staining of CK-18 and vimentin in endometrium before (above) and after (below) treatment (scale bar: 100 µm). (**C**) The mRNA expression of TNF-α, IL-4, IL-6, and IL-10 was assessed by quantitative real-time PCR (qRT-PCR). (**D**) The concentration of TNF-α, IL-4, IL-6, and IL-10 was detected by ELISA. (**E**) The mRNA expression of IGF-1, SDF-1, and TSP-1 was tested by qRT-PCR. *P < 0.05 vs Control group, ^#^*P* < 0.05 vs IUA group, and ^&^*P* < 0.05 vs Estrogen group; *n* = 3.

Next, the therapeutic effects of rrGH combined with estrogen on the endometrium of IUA rats were further investigated. First, immunohistochemistry (IHC) was performed to measure the expression of CK-18 and vimentin, serving as markers for endometrial, epithelial, and stromal cell regeneration, respectively. Compared with the Control group, CK-18 expression was downregulated, and vimentin level was significantly increased in the IUA group. A significant increase in the expression of CK-18 and a decrease in the expression of vimentin were observed in the rrGH, Estrogen, and Estrogen + rrGH groups compared to the IUA group, tentatively suggesting that the endometrial tissue began to regenerate. Notably, the Estrogen + rrGH group exhibited a more significant treatment effect compared to the Estrogen group (Fig. 2A and B). Next, the level of inflammatory factors in rats’ endometrium was examined. Compared with the Control group, the TGH, Estrogen, and Estrogen + rrGH groups significantly inhibited the expression of TNF-α and IL-6, while enhancing the expression of IL-4 and IL-10 in IUA rats. Among them, the Estrogen + rrGH group had the most significant role in regulating these inflammatory markers (Fig. 2C and D). In addition, the expression levels of insulin-like growth factor-1 (IGF-1), stromal cell-derived factor-1 (SDF-1), and thrombospondin-1 (TSP-1) secreted in rat endometrial tissues were significantly elevated in the TGH, Estrogen, and Estrogen + rrGH groups compared to the IUA group, with the effect of elevation being more pronounced in the Estrogen + rrGH group compared to the Estrogen group (Fig. 2E). These results indicated that the combined use of rrGH and estrogen inhibits endometrial inflammation and promotes endometrial regeneration in IUA rats.

### rrGH combined with estrogen regulates microbial diversity in the endometrium of IUA rats

**Fig 3:**
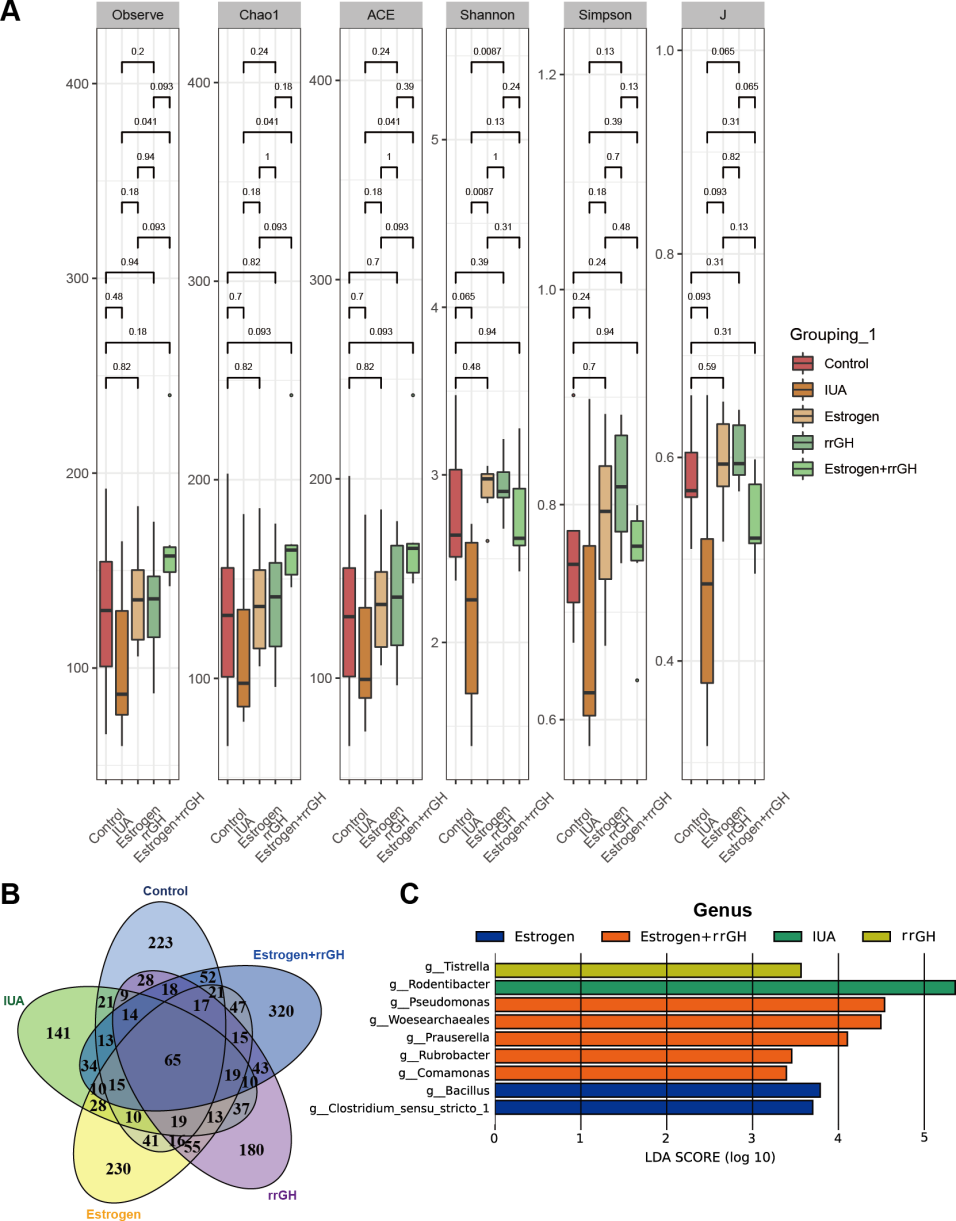
rrGH combined with estrogen regulates microbial diversity in the endometrium of IUA rats. (**A**) Alpha analysis of microbial changes. (**B**) Venn plot showing changes in microbial numbers. (**C**) Lefse analysis of microbiota at the genus level in the endometrium of rats.

16S rRNA sequencing was performed to determine the effects of rrGH combined with estrogen on endometrial microbiota. Indices such as observe, Chao1, ACE, Shannon, Simpson, and J were employed to evaluate the bacterial alpha diversity, richness, and evenness within the microbial communities. As depicted in Fig. 3A, the observe, Chao1, ACE, Shannon, Simpson, and J indices were diminished in the IUA group compared to the Control group, whereas these indices were further elevated by the administration of rrGH and estrogen, suggesting that rrGH and estrogen treatment modulated intragroup species diversity of rat endometrial microbiota (Fig. 3A). The Venn plot showed a decrease in the number of OTUs in the IUA group compared with the Control group. Conversely, the number of OTUs increased in the rrGH, Estrogen, and Estrogen + rrGH groups when compared to the IUA group, with the most significant increase in Estrogen + rrGH (Fig. 3B). The Lefse analysis presented the abundance of microbial taxa at the genus level (Fig. 3C). The IUA group exhibited a high enrichment of *p_Proteobacteria* and *g_Rodentibacter*. Notably, the Estrogen + rrGH group demonstrated higher abundances of *p_Nanoarchaeota*, along with *g_Rodentibacter*, *g_Pseudomonas*, *g_Woesearchaeales*, *g_Prauserella*, *g_Rubrobacter*, and *g_Comamonas*. Taken together, the combined use of GH and estrogen increased the microbial diversity in the endometrium of IUA rats.

### Effects of rrGH combined with estrogen on peripheral blood metabolism in IUA rats

**Fig 4:**
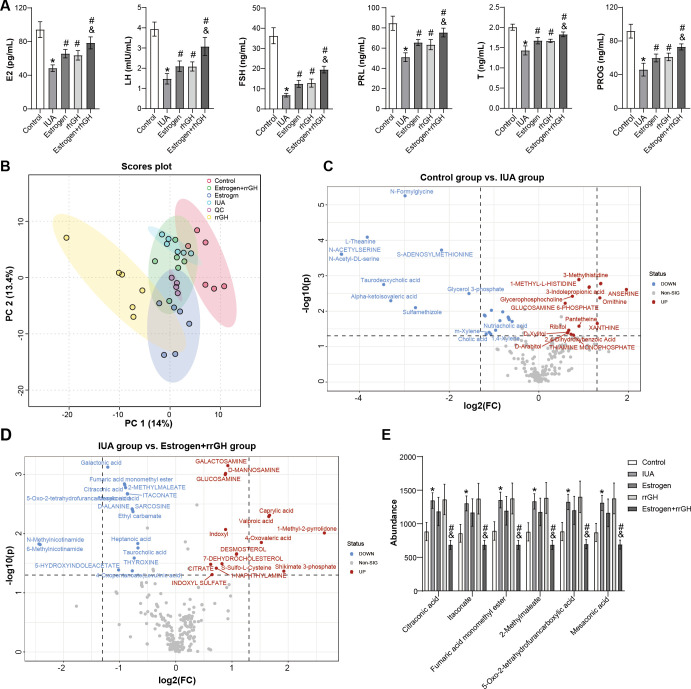
Effects of rrGH combined with estrogen on peripheral blood metabolism in IUA Rats. (**A**) The levels of FSH, LH, E2, P, T, and PRL in the peripheral blood of rats were measured by ELISA. (**B**) PCA grade outcomes of rat serum samples. (**C and D**) Volcano map showing differential metabolites. (**E**) Abundance analysis of differential metabolites. *P < 0.05 vs Control group, ^#^*P* < 0.05 vs IUA group, and ^&^*P* < 0.05 vs Estrogen group.

Next, the effects of rrGH combined with estrogen on serum metabolism were explored. The levels of critical serum hormones (FSH, LH, E2, P, T, and PRL) were first analyzed in the peripheral blood of rats. The results of the study showed that the levels of the above hormones were significantly elevated in the peripheral blood of IUA rats in the Estrogen group, rrGH group, and Estrogen + rrGH group. Notably, the levels of FSH, LHE2, P, T, and PRL were more significantly elevated in the Estrogen + rrGH group than in the Estrogen group (Fig. 4A). To further unravel the underlying metabolic mechanisms, an untargeted metabolomics analysis using rat serum samples was conducted, aiming to identify differential metabolites. Principal component analysis (PCA) was employed to assess the overall variations among groups. As depicted in Fig. 4B, the rrGH and Estrogen groups were distinctly separated from the Control and IUA groups in their data distributions, while the Estrogen + rrGH group exhibited partial overlaps with both, suggesting the complexity of its metabolic profile. Through meticulous analysis of metabolites, a total of 102 potential metabolic biomarker candidates were identified. To visually illustrate these differential metabolites, volcano plots were constructed, providing a clear representation of the trends in metabolite changes between groups. When compared to the Control group, 44 significantly different metabolites were identified in the IUA group, with 17 being significantly upregulated and 27 downregulated. In contrast to the IUA group, the Estrogen + rrGH group exhibited 32 differential metabolites, of which 15 were significantly upregulated and 17 were significantly downregulated (Fig. 4C and D). Figure 4E shows the metabolites shared among the metabolites significantly upregulated in the IUA group and significantly downregulated in the Estrogen + rrGH group. The metabolites such as citraconic acid, itaconate, fumaric acid monomethyl ester, 2-methylmaleate, mesaconic acid, and 5-oxo-2-tetrahydrofuran carboxylic acid were upregulated in the serum of IUA rats, whereas all of the above metabolites were significantly downregulated after combined estrogen and rrGH treatment (Fig. 4E). Collectively, these data demonstrated that the metabolism in serum of IUA rats was disordered, and rrGH combined with estrogen might improve IUA by regulating the metabolism in serum.

### rrGH combines with estrogen to alleviate endometrial fibrosis and promote β-catenin expression in IUA rats

**Fig 5:**
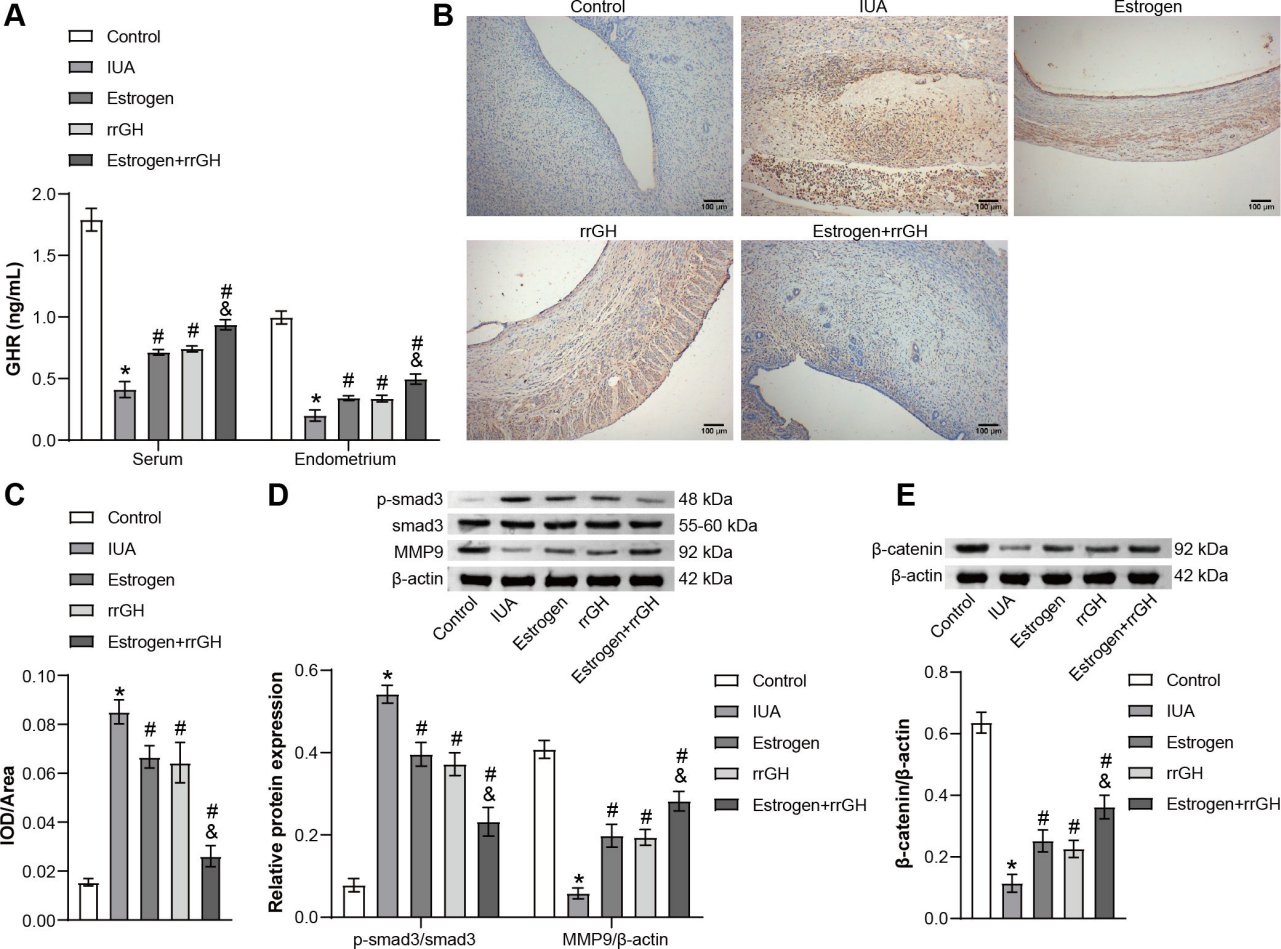
rrGH combined with estrogen to alleviate endometrial fibrosis and promote β-catenin expression in IUA rats. (**A**) The level of GHR in the peripheral blood and endometrium of rats was detected by ELISA. (**B and C**) IHC staining of TGF-β1 in the endometrium of rats (scale bar: 100 µm). (**D and E**) The expressions of p-smad3, smad3, MMP9, and β-catenin were assessed by WB. *P < 0.05 vs Control group, ^#^*P* < 0.05 vs IUA group, and ^&^*P* < 0.05 vs Estrogen group; *n* = 3.

Peripheral blood and endometrial GHR levels were first detected in rats. As shown in Fig. 5A, the GHR level in peripheral blood and endometrium was significantly decreased in the IUA group compared to the Control group, and the GHR level was increased following treatment with rrGH or estrogen. Notably, GHR levels were significantly higher in the Estrogen + IGH group compared to the estrogen group (Fig. 5A). Studies have reported that TGF-β1 is one of the important regulators that promote endometrial fibrosis (30). Our results showed that the expression of TGF-β1 in the endometrium of IUA rats was significantly upregulated compared to the Control group. Nevertheless, intervention with either rrGH or estrogen effectively suppressed TGF-β1 expression. Remarkably, the combined application of rrGH and estrogen exhibited a more prominent effect in reducing TGF-β1 expression than estrogen treatment alone (Fig. 5B and C). The above results suggested that estrogen and rrGH combination therapy can alleviate endometrial fibrosis. Further results showed that the p-smad3/smad3 ratio was significantly lower, and the expression of mmp9 and β-catenin was significantly higher in the endometrium of IUA rats in the Estrogen, rrGH, and Estrogen + rrGH groups compared with the IUA group. In addition, the p-smad3/smad3 ratio was significantly lower, and the expression of β-catenin was significantly higher in the Estrogen + rrGH group compared with that in the Estrogen group. The effect of the combination of estrogen and rrGH was more significant (Fig. 5D and E). Altogether, the above results validated that the combination of rrGH and estrogen could alleviate endometrial fibrosis and promote β-catenin expression.

### rrGH combines with estrogen to ameliorate IUA through modulation of endometrial microbiota

**Fig 6:**
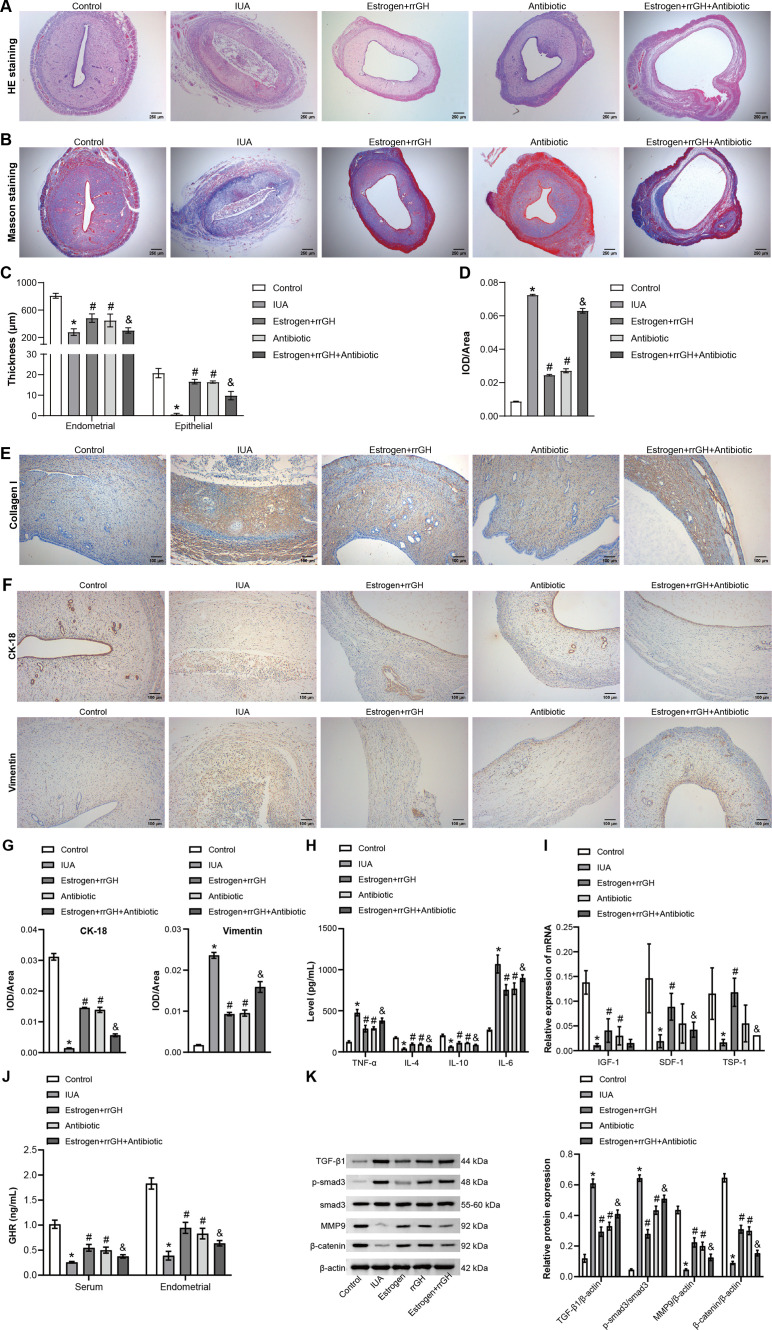
rrGH combined with estrogen to ameliorate IUA through the modulation of endometrial microbiota. (**A and B**) HE and Masson staining of the uterus before and after treatment (scale bar: 250 µm). (**C**) The thickness of the endometrium and epithelium before and after treatment. (**D and E**) IHC staining of collagen I expression in the endometrial tissue of IUA rats (scale bar: 100 µm). (**F and G**) IHC staining of CK-18 and vimentin in the endometrial tissue of IUA rats (scale bar: 100 µm). (**H**) The concentration of TNF-α, IL-4, IL-6, and IL-10 was detected by ELISA. (**I**) The mRNA expression of IGF-1, SDF-1, and TSP-1 was tested by quantitative real-time PCR. (**J**) The level of GHR in peripheral blood and endometrium of rats was detected by ELISA. (**K**) The expressions of p-smad3, smad3, MMP9, and β-catenin were assessed by WB. *P < 0.05 vs Control group, ^#^*P* < 0.05 vs IUA group, and ^&^*P* < 0.05 vs Estrogen + rrGH group; *n* = 3.

Next, it was explored in depth whether estrogen combined with rrGH treatment affects the IUA pathologic process by modulating the endometrial microbiota. The gut microbiome of IUA rats was first cleared by administering oral antibiotics to specifically evaluate the effect of antibiotic intervention on the efficacy of combined estrogen and rrGH treatment in the IUA rat model. It was observed that the degree of endometrial gland atrophy, endometrial and epithelial thickening, inflammatory cell infiltration, and abnormal collagen deposition were attenuated in the Estrogen + rrGH group and Antibiotic group compared with the IUA group and that the combined effect of estrogen and TGH was significantly attenuated by the antibiotic intervention (Fig. 6A through C). Second, collagen I expression was significantly reduced in both Antibiotic and Estrogen + rrGH groups compared to the IUA group, and collagen I expression was significantly elevated in the Estrogen + rrGH + Antibiotic group compared to the Estrogen + rrGH group. Antibiotic treatment reversed the inhibitory effect of estrogen combined with rrGH treatment on type I collagen expression in IUA rats (Fig. 6D and E). Furthermore, both antibiotic and rrGH combined with estrogen treatment effectively promoted the expression of CK-18 and inhibited the expression of vimentin in endometrial tissues of IUA rats. However, compared with the Estrogen + rrGH group, the expression of CK-18 was significantly reduced, and the expression of vimentin was significantly elevated in the Estrogen + rrGH + Antibiotic group. The antibiotic intervention reversed the therapeutic effect of estrogen combined with rrGH (Fig. 6F and G). The above results preliminarily indicated that antibiotics also had a therapeutic effect on IUA rats and that the antibiotic intervention cleared endometrial microorganisms, thus weakening the therapeutic effect of estrogen and rrGH to a certain extent. In addition, TNF-α and IL-6 levels were significantly higher, and IL-4, IL-10, IGF-1, SDF-1, and TSP-1 levels were significantly lower in the Estrogen + rrGH + Antibiotic group compared to the Estrogen + rrGH group. The antibiotic intervention reversed the effects of estrogen and rrGH on the regulatory ability of the above factors (Fig. 6H and I). Finally, GHR, MMP-9, and β-catenin levels were significantly higher, and TGF-β1 and Smad3 phosphorylation levels were lower in the Antibiotic and Estrogen + rrGH groups compared to the IUA group. Additionally, it was observed that antibiotics diminished the upregulatory effects of estrogen combined with rrGH treatment on GHR, MMP-9, and β-catenin, as well as their inhibitory effects on TGF-β1 expression and Smad3 phosphorylation (Fig. 6J and K). In conclusion, the therapeutic effects of rrGH and estrogen on IUA rats were attenuated by oral antibiotic cleansing of the intestinal microbiome in IUA rats, suggesting that estrogen in combination with rrGH treatment may ameliorate IUA by modulating endometrial microorganisms.

### Citraconic acid targets β-catenin to regulate TGF-β1-induced IUA model *in vitro*

**Fig 7:**
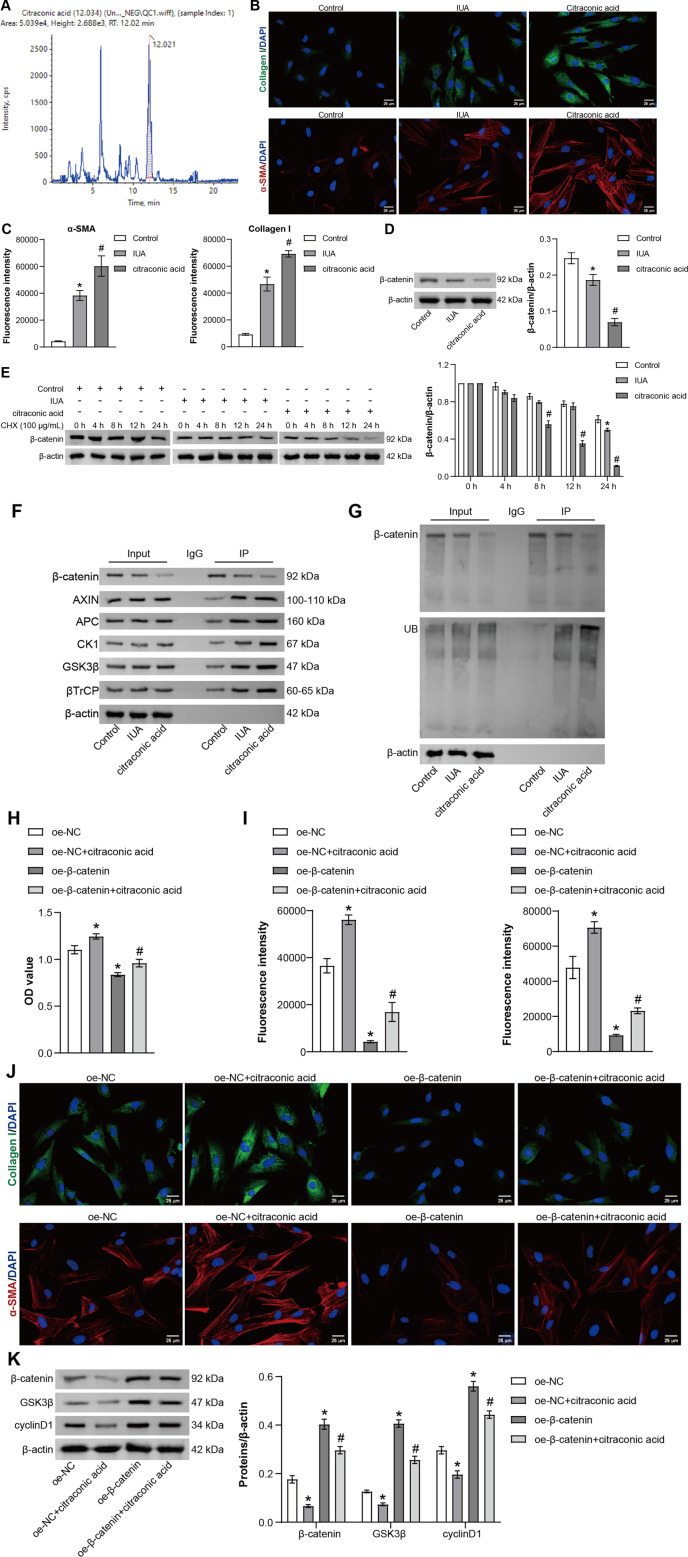
Citraconic acid targets β-catenin to regulate TGF-β1-induced IUA model *in vitro*. (**A**) The level of citraconic acid was determined by high-performance liquid chromatography-tandem mass spectrometry. (**B and C**) IF staining of α-SMA and collagen I in ESCs (scale bar: 25 µm). (**D**) The expressions of β-catenin were evaluated by WB. (**E**) The stability of β-catenin protein was detected by WB. *P < 0.05 vs Control group, ^#^*P* < 0.05 vs IUA group. (**F**) Co-immunoprecipitation (CO-IP) was used to detect the binding of β-catenin with AXIN, APC, CK1, GSK-3β, and β-TrCP. (**G**) CO-IP was used to detect the ubiquitination level of β-catenin. (**H**) Cell proliferation was detected by CCK-8. (**I and J**) IF staining of α-SMA and collagen I in ESCs. (**K**) The expressions of β-catenin, GSK-3β, and cyclin D1 were evaluated by WB. *P < 0.05 vs oe-NC group, ^#^*P* < 0.05 vs oe-β-catenin group; *n* = 3.

Finally, the impact of metabolites on the pathological process of IUA and its underlying mechanisms will continue to be elucidated. The binding of the differential metabolites and β-catenin was first analyzed by molecular docking. The binding energy between β-catenin and 2-methylmaleate, citraconic acid, and mesaconic acid was −5.4 kcal/mol, which was significantly higher than that with itaconate, fumaric acid monomethyl ester, and 5-oxo-2-tetrahydrofuran carboxylic acid (−5.2, −4.9, and −5.1 kcal/mol, respectively). The binding energies were less than −5 kcal/mol, indicating that the three differential metabolites were able to spontaneously bind well to the β-catenin protein. A lower binding energy (a more negative value) usually implies stronger binding. Therefore, β-catenin has a higher affinity for 2-methylmaleate, citraconic acid, and mesaconic acid (Fig. S1A). Next, the isolated ESCs were characterized, and positive CK19 and vimentin IF results were observed, indicating the successful isolation of ESCs (Fig. S1B). Then, after the establishment of *in vitro* cell models by TGF-β1-induced ESCs, the cells were treated with different concentrations (2.5–25 mM) of 2-methylmaleate, citraconic acid, and mesaconic acid, respectively, and it was found that the 10 mM concentration of citraconic acid had the most significant growth-promoting effect on the cells after 48 h of treatment (Fig. S1C). Therefore, 10 mM citraconic acid was selected for subsequent mechanistic studies. Figure 7A confirmed the presence of citraconic acid in the serum of rats receiving the combination treatment. Additionally, citraconic acid promoted the expression of α-SMA and collagen I in ESCs, suggesting that citraconic acid promotes fibrosis in ESCs (Fig. 7B and C), and citraconic acid also inhibited the expression of β-catenin and decreased the protein stability of β-catenin (Fig. 7D through G). The above results indicated that citraconic acid promotes the proliferation of ESCs and inhibits the expression of β-catenin. Further experiments showed that overexpression of β-catenin inhibited cell proliferation and decreased the expression of α-SMA and collagen I in cells while promoting the expression of GSK3β and cyclin D1 compared with the oe-NC group, and oe-citraconic acid showed the opposite effect. In contrast, compared with the oe-β-catenin group, cell viability, α-SMA, and collagen I were significantly elevated in the oe-β-catenin + oe-citraconic acid group, and the expression of GSK3β and cyclin D1 was decreased. The oe-citraconic acid reversed the oe-β-catenin action (Fig. 7H through K). Overall, it was emphasized that citraconic acid, as a metabolite, can affect the TGF-β1-induced IUA cell model by targeting the regulation of β-catenin, which in turn can play a therapeutic role.

## DISCUSSION

IUA is a common endometrial disease, usually caused by uterine injury, which can lead to a series of complications such as abnormal menstruation, recurrent abortion, secondary infertility, etc. (31, 32). The traditional therapy of IUA included hysteroscopic adhesiolysis and hormone therapy. Estrogen was a common hormone therapy in IUA, which promoted endometrial growth during preoperative treatment and enhanced endometrial regeneration during postoperative treatment (33). GH was widely used in assisted reproductive technology, which increased the thickness and receptivity of the endometrium (34). Therefore, GH might play a role in IUA treatment. In the present study, an IUA rat model was established to research the effect of GH combined with estrogen on IUA treatment. Compared to estrogen alone, the combination therapy significantly reduced collagen deposition and inflammatory cytokine levels in the endometrium, while promoting endometrial epithelial and stromal cell regeneration. In addition, factors such as IGF-1, SDF-1, and TSP-1 that were conducive to endometrial repair and regeneration were significantly increased in the endometrium of the combination group. In conclusion, our results suggested that GH effectively enhanced the therapeutic effect of estrogen. The combination of GH and estrogen was expected to be a new therapy for IUA treatment.

The microecological balance of the reproductive tract is essential for female health. There was growing evidence indicating that changes in the composition and distribution of the endometrial microbiome were associated with endometrial diseases, such as endometrial cancer, and infertility (35, 36). Chronic endometritis can cause endometrial microbial disorders (37). There were differences in the endometrial microbiome between IUA patients and infertile patients without intrauterine lesions, and the potential changes of endometrial microorganisms might be related to the occurrence of IUA (11). The 16S rRNA sequencing was conducted to investigate the effects of GH combined with estrogen on endometrial microbiota. Our results showed decreased α-diversity and a reduced number of OTUs in the IUA group, which further increased after the administration of rrGH and estrogen. Additionally, the IUA group exhibited high enrichment of *p_Proteobacteria* and *g_Rodentibacter*. The Estrogen + rrGH group displayed higher abundances of *p_Cyanobacteria* and *p_Nanoarchaeota*, along with *g_Rodentibacter*, *g_Pseudomonas*, *g_Woesearchaeales*, *g_Prauserella*, *g_Rubrobacter*, and *g_Comamonas*. It has been shown that Archaea are not only found in the deep sea but also in many different ecosystems (38), such as the gastrointestinal tract, lungs, and skin (39). Archaea play a significant role in natural ecosystems and the human body (38). The study by Xue et al*.* (40) reported that thiamine supplementation significantly increased Crenarchaeota, Nanoarchaeota, and the Candidatus phyla but decreased Thaumarchaeota compared with high-concentrate diet treatment in cows. Furthermore, the presence of nanoarchaea in human oral microorganisms was first reported by Hassani et al*.* (41) who utilized specific PCR-based assays to detect a novel nanoarchaea, *Nanopusillus massiliensis*. A reliable PCR-based assay was proposed to determine the presence of methane nanoarchaea in intestinal biopsy samples, and they also validated it by 16S rRNA gene amplification of archaea. Thus, it was concluded that *p*_*Nanoarchaeota* not only exists in the deep sea but is also realistically detected in our experiments. Additionally, the effect of the Estrogen + rrGH group and antibiotic treatment alone was similar, which suggests that the presence of our bacteria may play a negative role and thus aggravate the degree of endometrial damage. The antibiotic treatment cleared microorganisms and weakened the effect of the Estrogen + rrGH combination treatment, which suggests that endometrial microorganisms play an active role in the treatment of IUAs with Estrogen + rrGH. Therefore, we speculated that endometrial microbes may play a positive or negative role depending on the specific situation and suggest that the potential role of the microbial environment on intrauterine adhesion fibrosis needs to be appropriately considered during clinical treatment according to the treatment strategy. Our findings are potentially instructive for the clinical management of intrauterine adhesion fibrosis, especially the combination of antibiotics.

Research shows that the serum metabolism of IUA rats was abnormal (42). Therefore, non-targeted metabolomics was performed to evaluate the effects of GH combined with estrogen on serum metabolites and related metabolic pathways in IUA rats. Herein, a total of 253 metabolites were identified, and 102 potential biomarkers were obtained by further analysis of metabolites. Besides, the levels of metabolites such as citraconic acid, itaconate, fumaric acid monomethyl ester, 2-methylmaleate, mesaconic acid, and 5-oxo-2-tetrahydrofuran carboxylic acid were increased, which was decreased by the combination treatment. These metabolites may be potential metabolic characterizations and biomarkers for tracking and evaluating the effects of Estrogen + rrGH treatment. Further molecular docking technology revealed that β-catenin and differential metabolites like citraconic acid, 2-methylmaleate, and mesaconic acid have high affinity, implying that these metabolites may play key roles in the regulation of the β-catenin signaling pathway.

The most important pathological feature of IUA was intrauterine fibrosis (43). TGF-β1 was an important fibrogenic mediator, which promoted endometrial fibrosis through the Smad pathway (44). GH combined with estrogen significantly reduced the expression of TGF-β1 and p-smad3 in the endometrium. Moreover, a vast literature reported that targeting the Wnt/β-catenin signaling pathway observably improved endometrial fibrosis (45, 46). In our study, GH combined with estrogen markedly upregulated the expression of MMP9 and β-catenin, indicating that the combination treatment relieved endometrial fibrosis in IUA rats by activating the β-catenin signaling pathway. Our cell experiments confirmed our conclusion once again. In the TGF-β1-induced IUA cell model, treatment with TGF-β1 promoted the expression of α-SMA and collagen I but reduced the expression level and stability of β-catenin protein. The effect of TGF-β1 was further strengthened by citraconic acid intervention. Additionally, we found that overexpression of β-catenin could reduce the expression of α-SMA and collagen I and increase the expression of GSK-3β and cyclin D1 proteins. However, these changes were reversed by the intervention of citraconic acid.

However, there are some limitations to our study: clinical treatment requires long-term interventions, and animal experiments have short treatment cycles, so we did not explore estrogen + rrGH on endometrial damage in rats in the long term. In our *in vitro* experiments, we only explored citraconic acid, which is the most significant in promoting the proliferation of endometrial stromal cells, and did not explore the possible mechanisms of action of the remaining metabolites such as 2-methylmaleate and mesaconic acid. We plan to further investigate them in future experiments. Additionally, the 16S sequencing used in our study may not be as well-established as macro-genomic or triple sequencing.

### Conclusion

In conclusion, our research indicated that GH combined with estrogen relieved endometrial fibrosis by regulating the metabolite citraconic acid of the microbial microbiome to target the β-catenin pathway. Furthermore, compared with estrogen alone, the combination therapy for IUA treatment was obviously better, suggesting that the combination of GH and estrogen may be a promising treatment strategy.

## Data Availability

The raw data of 16S sequencing have been uploaded to the SRA database (PRJNA934667). Additional original data can be requested from the corresponding author when necessary.

## SUPPLEMENTAL MATERIAL

Figure S1 msystems.01692-24-s0001.tif Citraconic acid targets β-catenin to regulate TGF-β1-induced IUA model *in vitro*.

Supplemental material msystems.01692-24-s0002.docx Molecular docking information and Table S2.

Table S1 msystems.01692-24-s0003.xlsx Results of untargeted metabolomics analysis of serum metabolites after treatment of IUA mice.

ASM does not own the copyrights to Supplemental Material that may be linked to, or accessed through, an article. The authors have granted ASM a non-exclusive, world-wide license to publish the Supplemental Material files. Please contact the corresponding author directly for reuse.
