# Supplementary figures and images for: Growth hormone combined with estrogen improves intrauterine adhesion fibrosis by downregulating endometrial microbial citraconic acid to target β-catenin protein
Source: mSystems. 2025 Jun 5;10(7):e01692-24. doi: 10.1128/msystems.01692-24 (PMC12282089; doi:10.1128/msystems.01692-24)

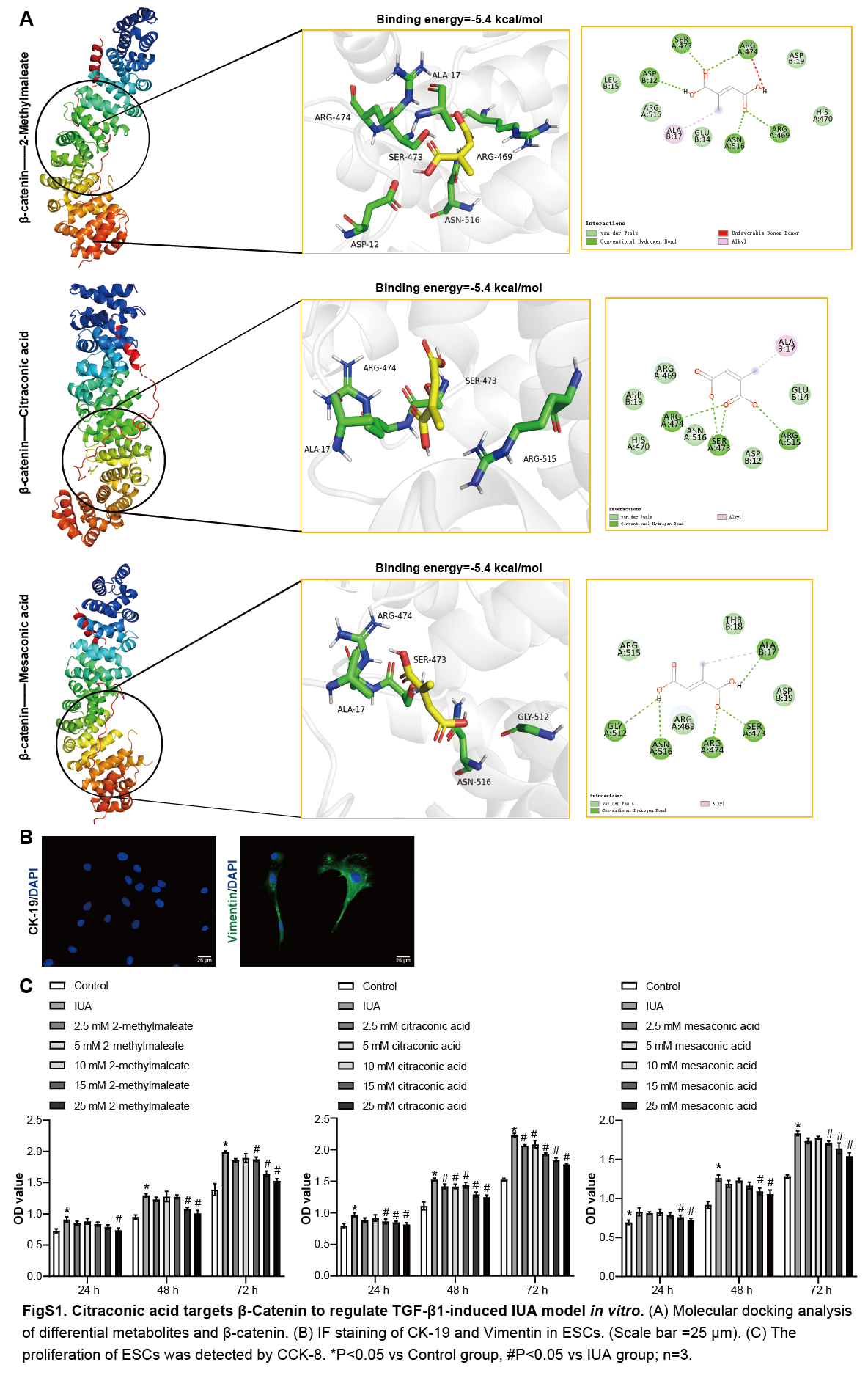

Supplement: Figure S1 — Citraconic acid targets β-catenin to regulate TGF-β1-induced IUA model in vitro. [file msystems.01692-24-s0001.tif]
