# Supplementary material for: Growth hormone combined with estrogen improves intrauterine adhesion fibrosis by downregulating endometrial microbial citraconic acid to target β-catenin protein
Source: mSystems. 2025 Jun 5;10(7):e01692-24. doi: 10.1128/msystems.01692-24 (PMC12282089; doi:10.1128/msystems.01692-24)
Supplement: Supplemental material — Molecular docking information and Table S2. [file msystems.01692-24-s0002.docx]

Structure acquisition and preprocessing of protein crystals: beta-catenin protein was downloaded from the PDB database (https://www.rcsb.org/) and subsequently the protein was preprocessed by the pymol software to remove water molecules, hydrogenation as well as a charge from it. Then docking of beta-catenin protein and small molecules (name) was performed using autodock vina. After selecting the prepared ligand compound structure and the prepared macromolecule as the receptor and saving it in pdbqt format, the centers of the docked boxes were selected that the centers for the points were set as (mesaconic acid: center_x = 37.768, center_y = -19.213, center_z = 2.768, size_x = 46.5, size_y = 47.25，size_z = 47.25; citraconic acid: center_x = 37.768, center_y = -19.213, center_z = 2.768, size_x = 46.5, size_y = 47.25, size_z = 47.25; 2-methylmaleate: center_x = 37.768, center_y = -19.213, center_z = 2.768, size_x = 46.5, size_y = 47.25, size_z = 47.25), with spacing set to 0.375.

Amino acid residue list for the interaction of beta-catenin protein with small molecules (Supplementary Table 2)

|  |  | Interacting amino acid residues | | |
| --- | --- | --- | --- | --- |
| small molecules | Binding energy (kcal/mol) | hydrogen bonds | hydrophobic interactions | Van der Waals forces |
| mesaconic acid | -5.4 | A chain: GLY 512, ASN 516, ARG 474, SER 473;  B chain: ALA 17 | B chain: ALA 17 | A chain: ARG 515 ARG 469  B chain: ASP 19 ARG 469 |
| citraconic acid | -5.4 | A chain: ARG 474 SER 473 ARG 515 | B chain: ALA 17 | A chain: ARG 469 HIS 470 ASN 516  B chain: ASP 19 ASP 12 GLU 14 |
| 2-methylmaleate | -5.4 | A chain: SER 473 ARG 474 ARG 469 ASN 516  B chain: ASP 12 | B chain: ALA 17 | A chain: ARG 515 HIS 470  B chain: LEU 15 GLU 14 ASP 19 |
